# Facial vascularized composite allotransplantation: Mapping global research trends and scientific impact

**DOI:** 10.1016/j.jpra.2026.08.004

**Published:** 2026-08-19

**Authors:** André S. Alves, Tobias Niederegger, Leonard Knoedler, Curtis L. Cetrulo, Alexandre G. Lellouch

**Affiliations:** a Division of General Surgery, Geneva University Hospitals, 1205 Geneva, Switzerland; b University of Heidelberg, Medical Faculty Heidelberg, Heidelberg, Germany; c Charité–Universitätsmedizin Berlin, corporate member of Freie Universität Berlin and Humboldt-Universität zu Berlin, Department of Oral and Maxillofacial Surgery, Mittelallee 2, 13353 Berlin, Germany; d Division of Plastic and Reconstructive Surgery, Cedars-Sinai Medical Center, Los Angeles, CA, USA

**Keywords:** Facial transplantation, Vascularized composite allotransplantation, Tissue engineering, Transplant, VCA

## Abstract

**Background:**

Facial vascularized composite allotransplantation (VCA) has emerged as a transformative reconstructive option for patients with severe facial disfigurement. Since its clinical introduction, research has expanded substantially, encompassing surgical innovation, immunology, ethical considerations, and long-term outcomes. This study aimed to analyze the global scientific landscape of facial VCA and identify key contributors, landmark publications, and emerging research trends through a bibliometric approach.

**Methods:**

A bibliometric analysis of the PubMed database was conducted in March 2026, including publications from 1979 to 2026. A total of 457 articles met the inclusion criteria. Bibliometric indicators such as annual scientific output, authorship patterns, institutional productivity, country contributions, collaboration networks, and keyword trends were analyzed using VOSviewer and the Bibliometrix R package.

**Results:**

Scientific production increased markedly after 2009, peaking around 2019 with sustained activity thereafter. The most productive authors were Dr Pomahac B. (n = 81), Dr Rodriguez E.D. (n = 48), and Dr Siemionow M. (n = 28). Harvard Medical School was the leading institution, and the United States dominated global output. Plastic and Reconstructive Surgery was the most productive journal. The most cited publication was “Facial transplantation: the first 9 years” (308 citations). Keyword analysis revealed a shift toward emerging topics including antibody-mediated rejection, chronic rejection, biomarkers, proteomics, C4d, and donor-specific antibodies.

**Conclusions:**

Facial VCA research has expanded rapidly, driven by high-volume centers and influential studies. Emerging trends emphasize advanced immunologic monitoring and molecular approaches, which may improve long-term graft survival and clinical outcomes.

## Introduction

Severe facial defects resulting from trauma, burns, oncologic resection, or congenital conditions remain among the most challenging problems in reconstructive surgery. While conventional autologous reconstruction can provide structural restoration and partial functional recovery, it is often insufficient to achieve optimal aesthetic integration, sensory recovery, and complex motor function in extensive defects. In this context, vascularized composite allotransplantation (VCA) has emerged as a transformative reconstructive strategy, enabling the transplantation of multiple tissue types, including skin, muscle, bone, nerves, and vasculature, as a single functional unit.^1^, ^2^, ^3^ Facial transplantation, as a highly specialized form of VCA, offers the unique potential to restore both form and function in patients with severe facial disfigurement, significantly improving quality of life.^4^^,^^5^

Since the first partial face transplantation performed in 2005, the field has evolved rapidly, with increasing numbers of procedures performed worldwide since 2010 and continuous refinements in surgical techniques and perioperative management.^6^^,^^7^ However, despite these advances, long-term graft survival remains limited by immunologic challenges, particularly acute and chronic rejection, as well as complications related to lifelong immunosuppression.^8^^,^^9^ Consequently, ongoing research has focused on better understanding the mechanisms of graft rejection and improving strategies for graft monitoring and long-term outcomes.

In parallel with these clinical and scientific developments, the body of literature on facial VCA has expanded substantially over the past two decades.^10^^,^^11^ Research efforts span a wide range of domains, including surgical innovation, transplant immunology, regenerative medicine, ethical considerations, and long-term functional and psychosocial outcomes.^12^^,^^13^ As the field continues to evolve, it becomes increasingly important to understand not only clinical progress but also the structure and direction of scientific research that underpins these advances.

Bibliometric analysis provides a quantitative framework to evaluate scientific production, identify key contributors, and map the evolution of research topics within a given field. By analyzing publication trends, authorship networks, institutional productivity, and thematic developments, bibliometric methods can offer valuable insight into the maturation of emerging disciplines and highlight areas of growing scientific interest. Such analyses can help identify shifts toward novel research directions, including immunologic monitoring, molecular characterization of rejection, and technological innovation.

Despite the growing body of literature, comprehensive bibliometric evaluations of facial vascularized composite allotransplantation remain limited. A detailed mapping of the scientific landscape is essential to better understand the development of the field, identify leading institutions and influential publications, and highlight emerging trends that may shape future research and clinical practice. Therefore, the aim of this study was to perform a bibliometric analysis of the global scientific literature on facial VCA, evaluating publication trends, key authors and institutions, geographic distribution of research output, and evolving research themes, with particular attention to recent advances in immunologic and molecular characterization of graft outcomes.

## Methods

### Study design and data source

A bibliometric analysis was conducted to evaluate the scientific literature on facial VCA. The PubMed database was selected as the primary source due to its comprehensive coverage of biomedical and surgical research.

### Search strategy

A systematic search was performed to identify relevant publications from database inception through March 2026. The search strategy included combinations of keywords and Medical Subject Headings related to facial transplantation, including: “facial transplantation,” “face transplant,” “vascularized composite allotransplantation,” “VCA,” and related terms. Boolean operators (“AND,” “OR”) were used to refine the search and maximize sensitivity (Supplementary Material 1.).

### Eligibility criteria

The bibliometric analysis was based on the publications retrieved using the predefined PubMed search strategy. No additional systematic study selection or full-text eligibility assessment was performed. The specificity of the search query was intended to identify the body of literature most relevant to facial vascularized composite allotransplantation for bibliometric analysis. The bibliometric analysis was restricted to English-language publications indexed in PubMed. This approach was chosen to ensure consistency in metadata extraction and analysis, as English represents the predominant language of internationally indexed biomedical publications within PubMed. Although the study aimed to characterize the global research landscape of facial vascularized composite allotransplantation, the term "global" refers to the geographical distribution of research contributions rather than the inclusion of publications in all languages.

### Data extraction

Relevant bibliographic data were extracted from each included publication, including publication year, authorship, institutional affiliation, country of origin, journal source, and citation metrics. Author names and institutional affiliations were standardized to minimize inconsistencies due to variations in indexing.

### Bibliometric analysis

Bibliometric indicators were analyzed to assess scientific productivity, impact, and collaboration patterns. Annual publication output was evaluated to identify temporal trends. Author productivity and institutional contributions were analyzed to identify key contributors in the field. Institutional productivity was quantified as the number of institutional affiliation occurrences within the retrieved publications rather than the number of unique articles, as a single publication may include multiple institutional affiliations. Country-level analysis was performed to assess global research distribution. Source analysis was conducted to determine the most productive journals publishing facial VCA research. Citation analysis was used to identify highly cited publications and assess their impact on the development of the field.

### Network and trend analysis

Collaboration networks between authors, institutions, and countries were analyzed to evaluate patterns of scientific cooperation. Keyword co-occurrence analysis was performed to identify major research themes and emerging topics. Trend analysis focused on identifying recently increasing terms to highlight evolving areas of interest, including immunologic monitoring, biomarker development, and advanced technologies.

### Data analysis and visualization

Data were analyzed and visualized using RStudio (Version 2024.09.0) and bibliometric software tools, including the Bibliometrix R package (Version 5.2.1) and VOSviewer (Version 1.6.20), enabling the generation of collaboration maps, trend analyses, and productivity graphs. Fig. 2 was generated using the Bibliometrix package in R and represents the cumulative annual publication output, illustrating the temporal growth of the facial VCA literature over the study period rather than a predictive statistical model. Descriptive statistics were used to summarize the data.

## Results

### Publication output and temporal trends

**Fig. 1:**
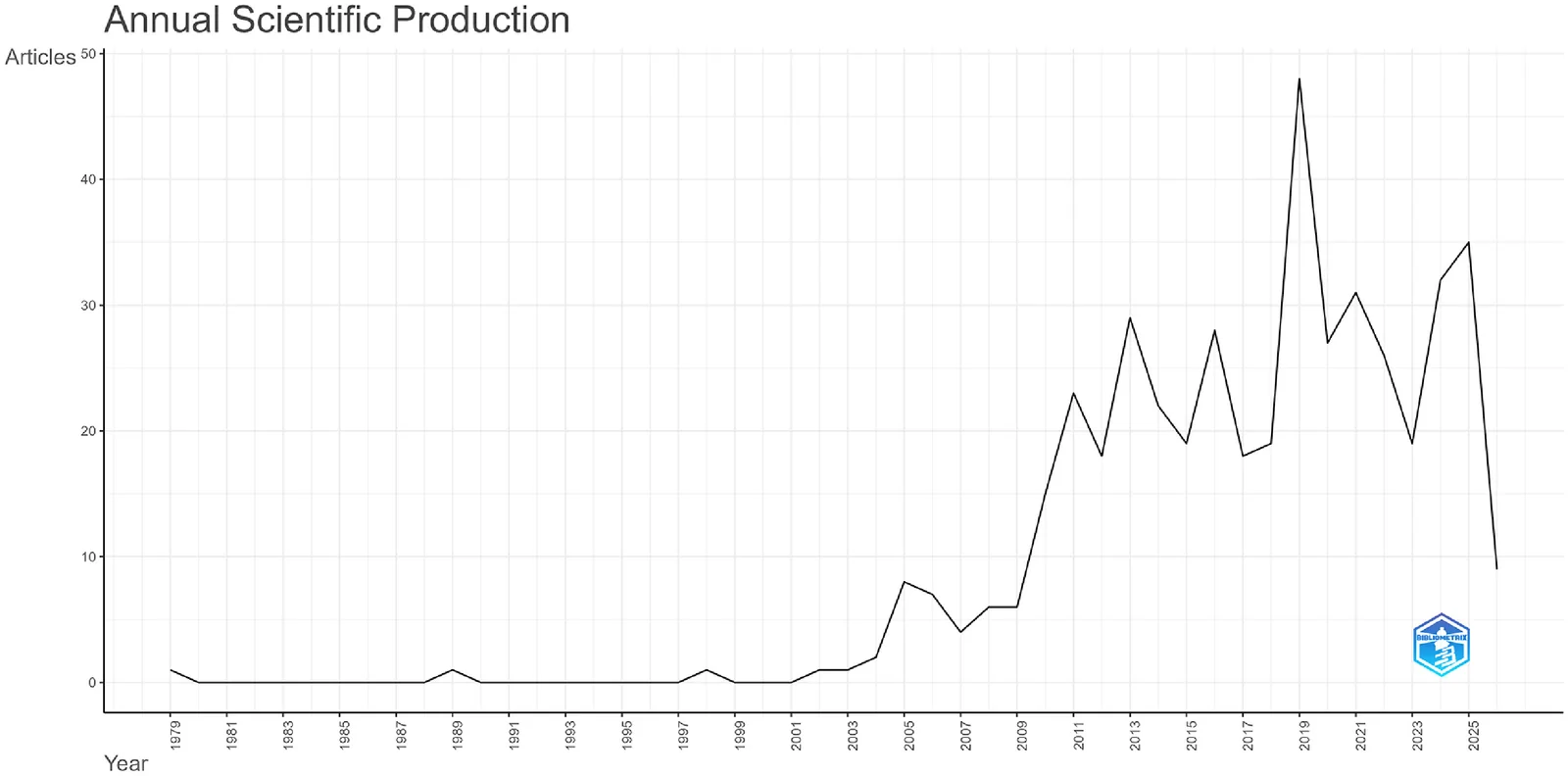
Annual scientific production of publications related to facial vascularized composite allotransplantation (1979–2026).

**Fig. 2:**
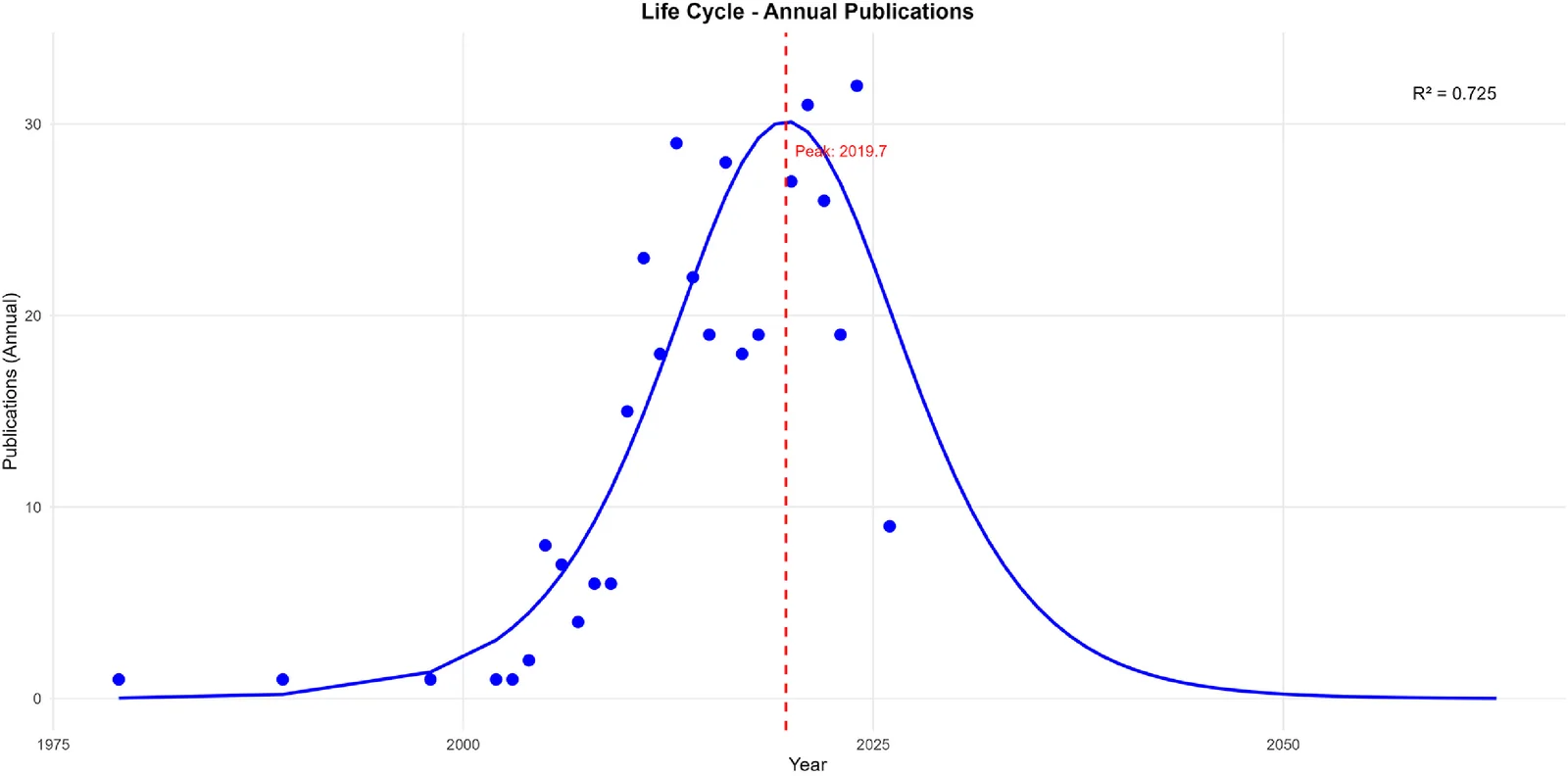
Life Cycle of publications on facial vascularized composite allotransplantation over time.

A total of 457 publications related to facial vascularized composite allotransplantation were identified between 1979 and 2026. Scientific production remained limited until the early 2000s, followed by a progressive increase after the first clinical face transplantation procedures (Fig. 1). A marked growth in publications was observed after 2009. Annual scientific production peaked around 2019, with sustained publication activity in subsequent years (Fig. 2). The cumulative growth curve demonstrated a steady and continuous increase in the number of publications over time.

### Country-Level scientific production

**Fig. 3:**
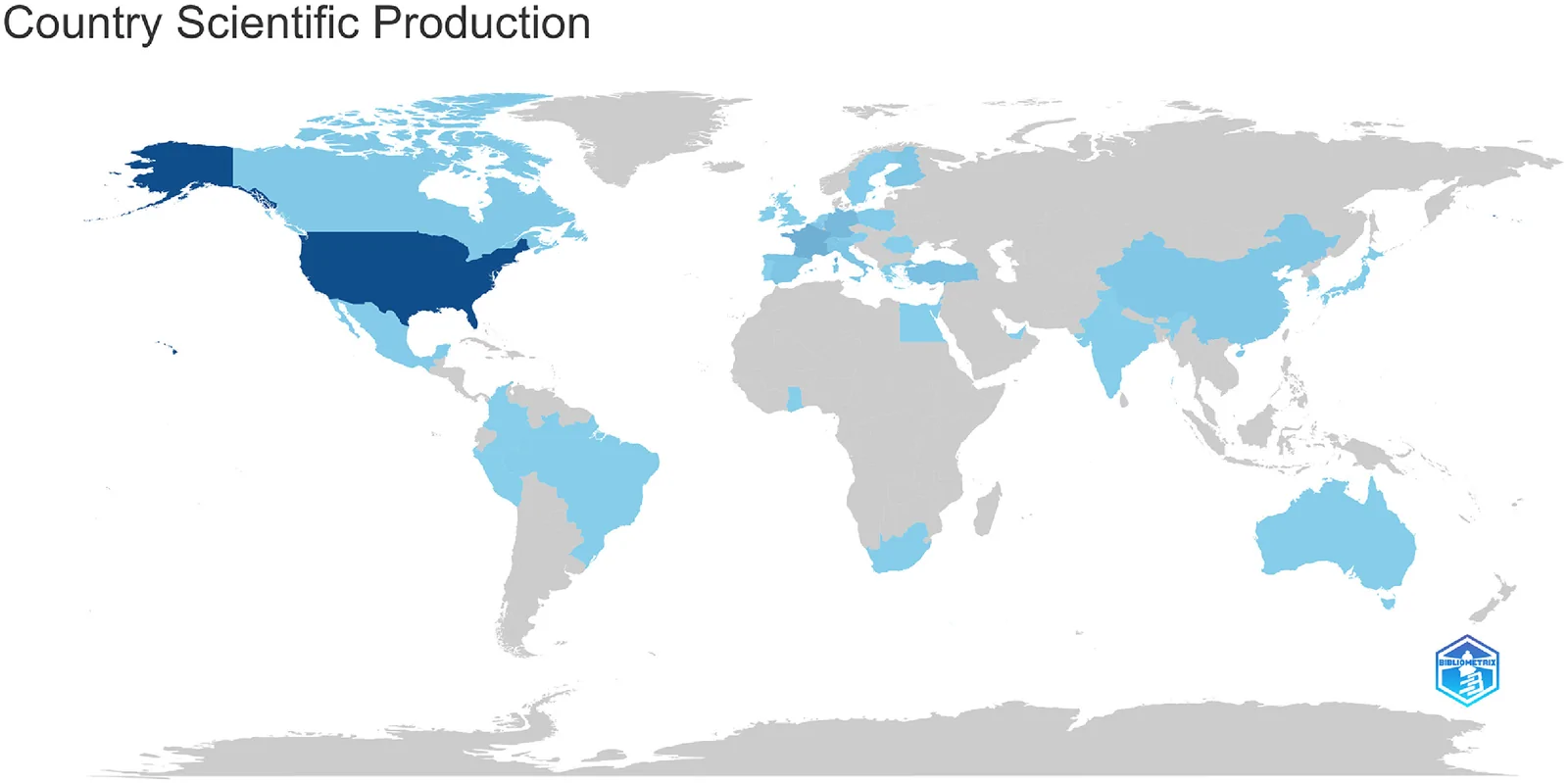
Country-level scientific production in facial vascularized composite allotransplantation research.

At the country level, the United States demonstrated the highest research output, followed by several European countries including France, Germany, and Spain, with additional contributions from Turkey, Canada, China, and Australia (Fig. 3). In the geographic visualization, darker color intensity represents greater publication output, illustrating the concentration of facial VCA research within a limited number of countries. The collaboration network analysis revealed strong international partnerships among institutions involved in vascularized composite allotransplantation research.

### Institutional contributions

**Fig. 4:**
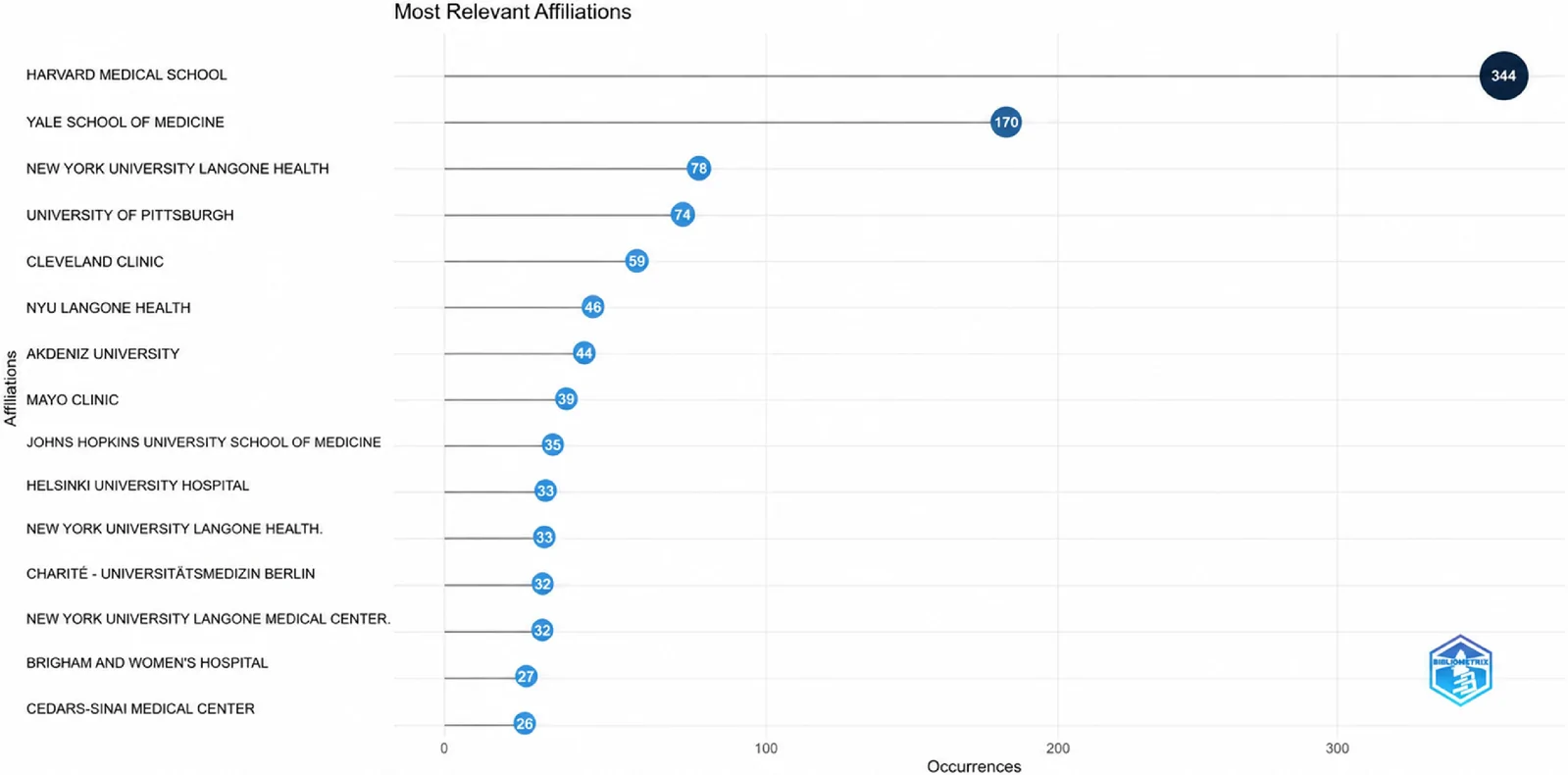
Most productive institutional affiliations contributing to facial vascularized composite allotransplantation research.

**Fig. 5:**
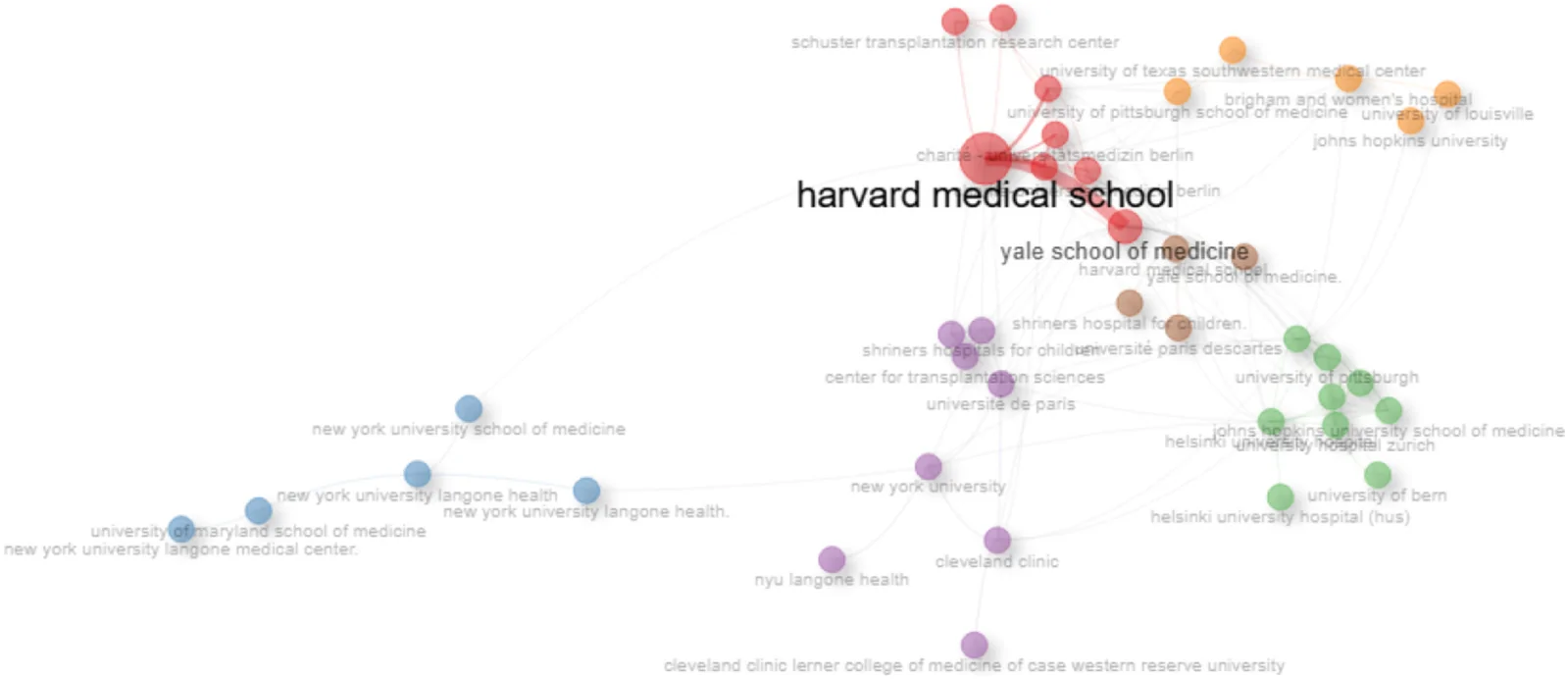
International collaboration network between institutions involved in facial vascularized composite allotransplantation research.

Institutional analysis identified Harvard Medical School as the most productive affiliation in the field, with a markedly higher publication output compared to all other institutions (Fig. 4). This was followed by Yale School of Medicine and New York University Langone Health, along with other leading centers such as the University of Pittsburgh and the Cleveland Clinic. Because individual publications may include multiple institutional affiliations, these values represent affiliation occurrences rather than unique publications and therefore reflect institutional participation in the literature rather than exclusive article counts. Beyond productivity, the collaboration network revealed a highly interconnected structure centered around these key institutions (Fig. 5). In this network visualization, node size is proportional to the number of collaboration occurrences, colors represent institutional collaboration clusters identified by the clustering algorithm, and edge thickness reflects the strength of collaborative relationships between institutions. Harvard Medical School functioned as a major hub, maintaining extensive collaborative links with multiple national and international partners. Secondary clusters were observed around Yale School of Medicine, indicating strong intra- and inter-institutional collaboration patterns. Additional regional clusters, particularly among European institutions, further highlighted the presence of geographically structured research networks.

### Source analysis

**Fig. 6:**
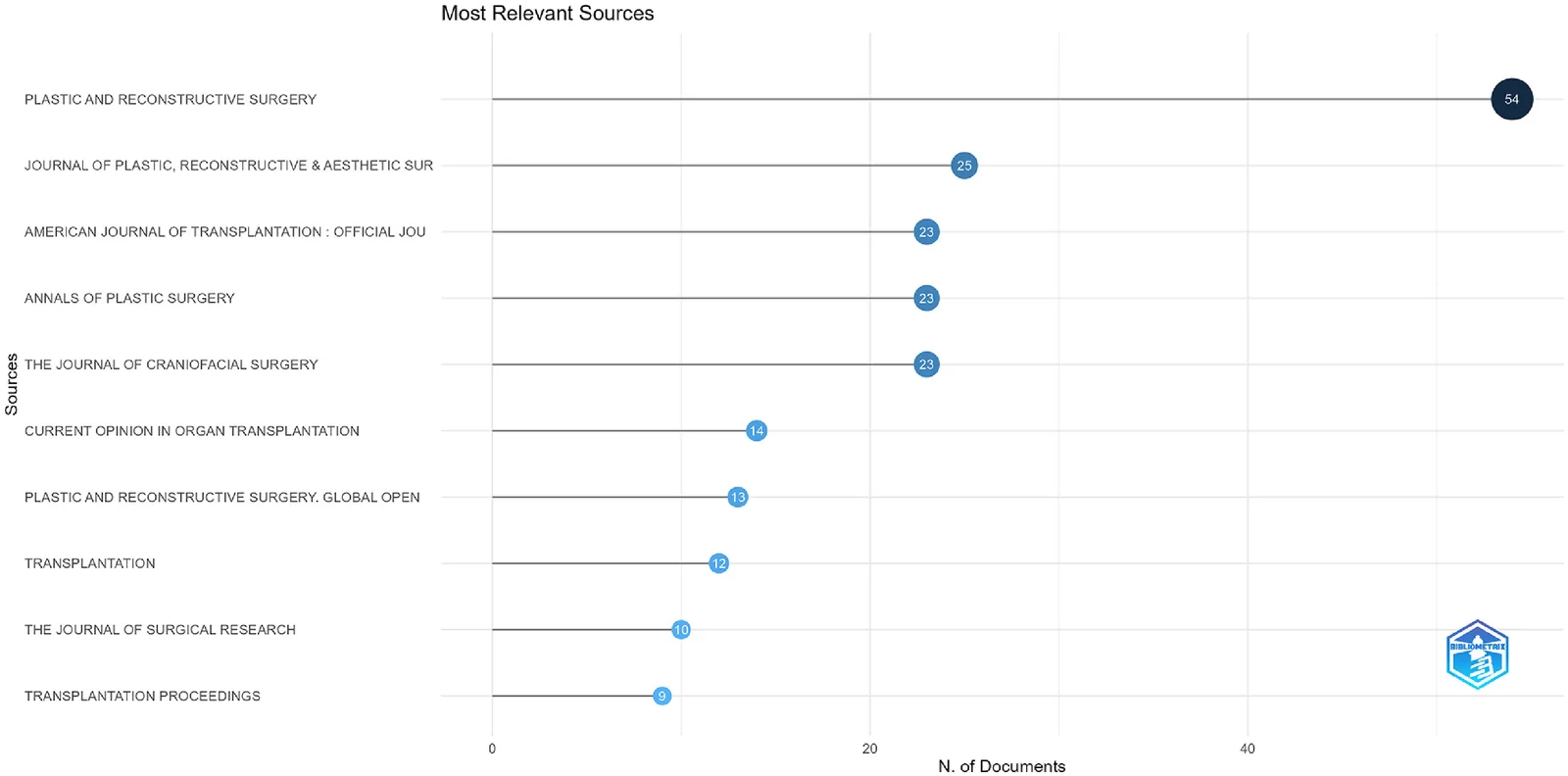
Most productive journals publishing research on facial vascularized composite allotransplantation.

**Fig. 7:**
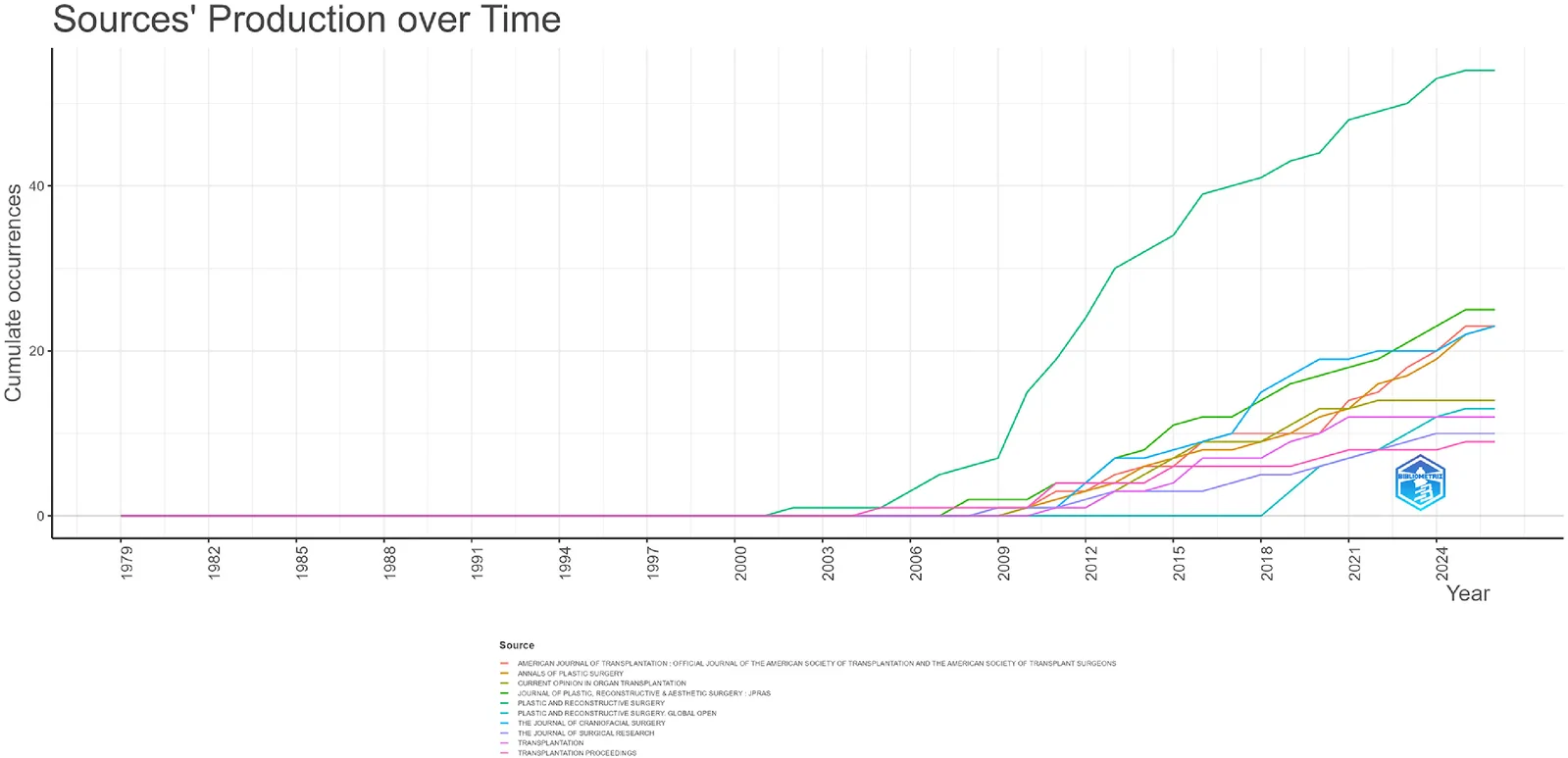
Source dynamics showing the evolution of journal contributions over time.

Among scientific journals, *Plastic and Reconstructive Surgery* was identified as the most productive source, publishing the highest number of articles related to facial vascularized composite allotransplantation (Fig. 6). Analysis of publication dynamics over time demonstrated a progressive increase in cumulative output across leading journals, particularly after 2009, corresponding with the expansion of clinical face transplantation programs (Fig. 7). *Plastic and Reconstructive Surgery* showed the most pronounced and sustained growth, maintaining a leading position throughout the study period. Other journals exhibited delayed but steady increases in publication activity, highlighting the gradual dissemination of VCA research across multiple scientific platforms.

### Most productive authors

**Fig. 8:**
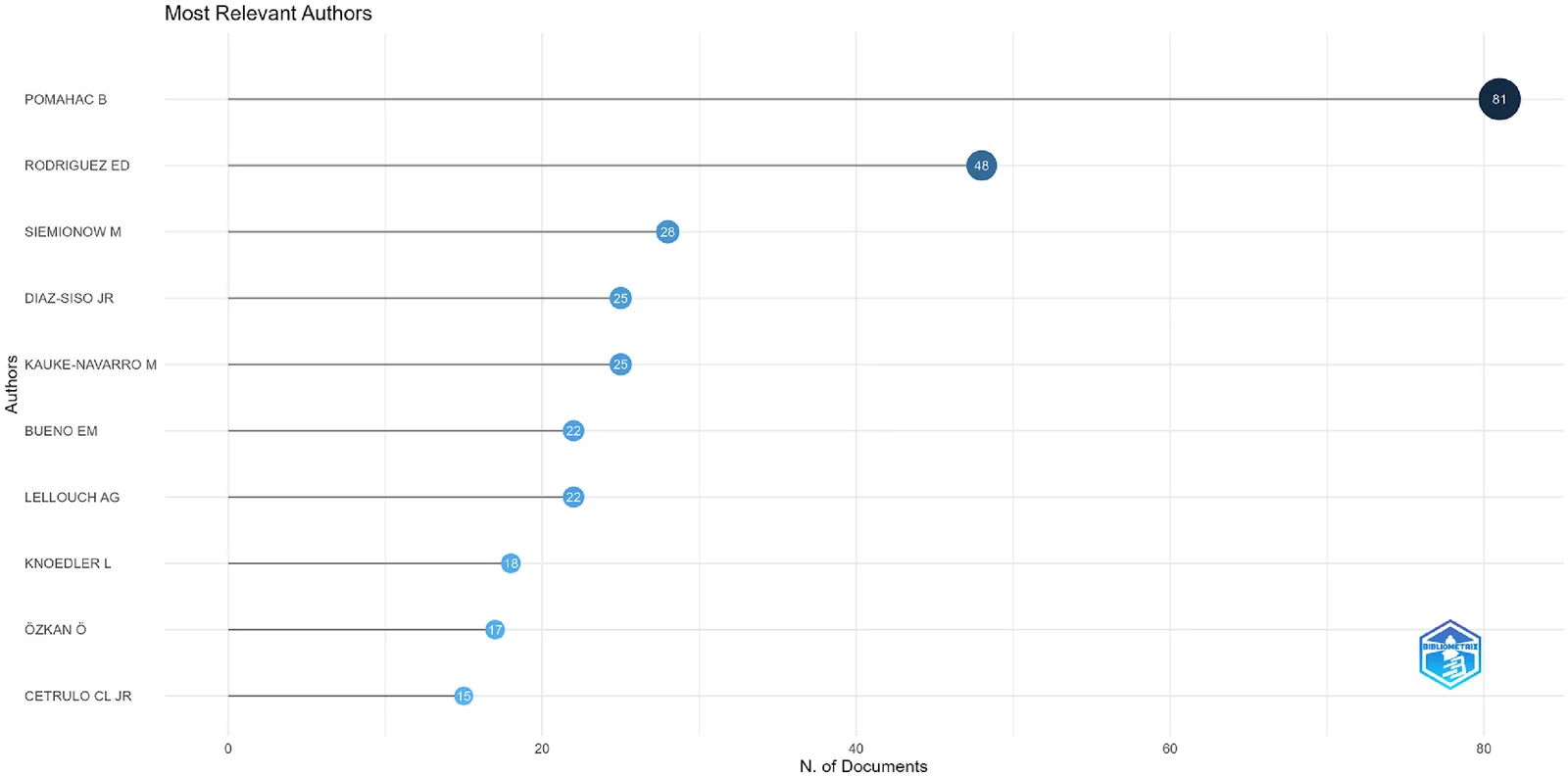
Most productive authors in facial vascularized composite allotransplantation research.

**Fig. 9:**
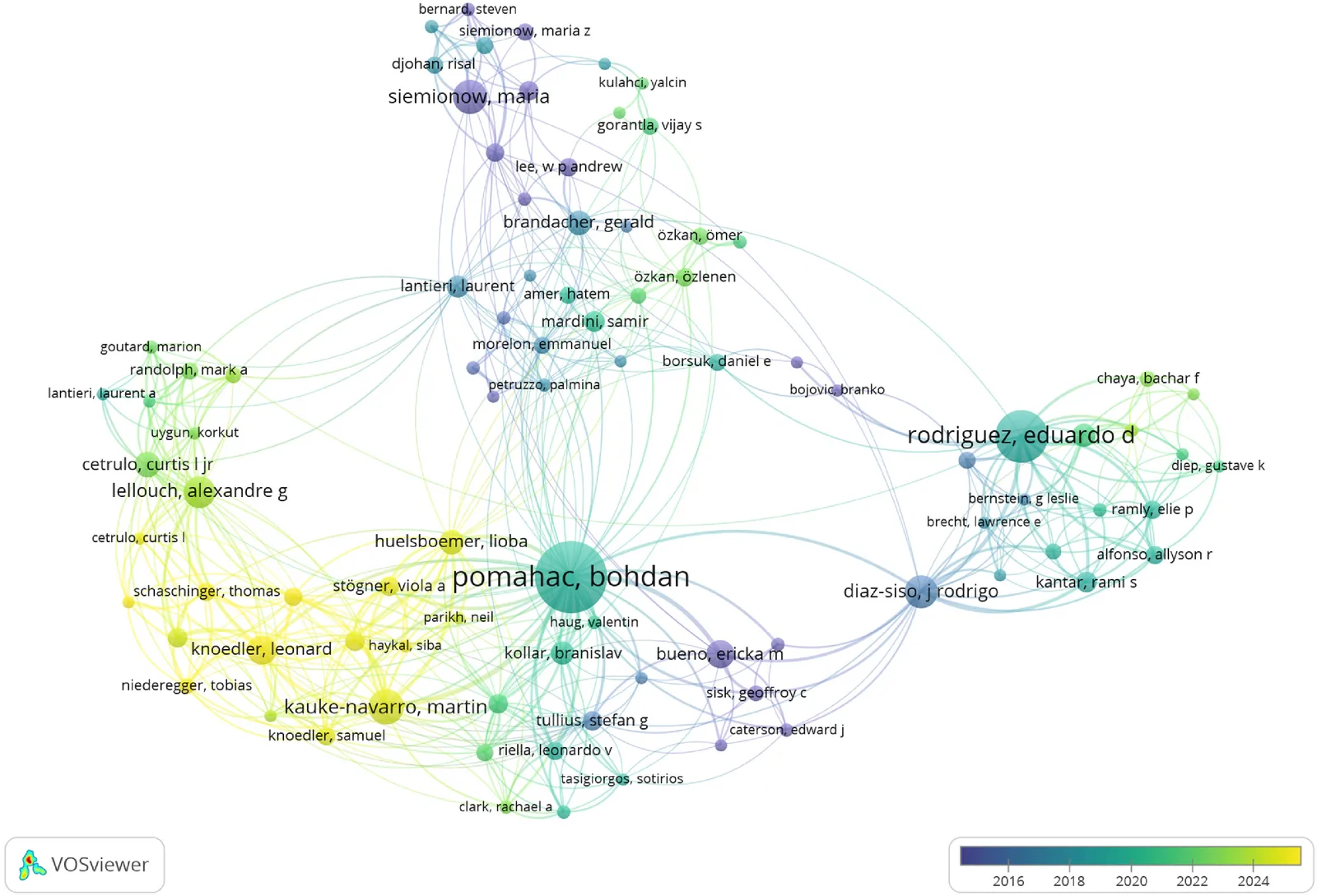
International collaboration network between authors involved in facial vascularized composite allotransplantation research.

Analysis of author productivity identified Dr Pomahac Bohdan (n = 81), Dr Rodriguez Eduardo D. (n = 48), Dr Siemionow Maria (n = 28), Dr Diaz-sisoRodrigo J (n = 25), Dr Kauke-Navarro Martin (n = 25), Dr Bueno Ericka M. (n = 22), Dr Lellouch Alexandre G. (n = 22), Dr Knoedler Leonard (n = 18), Dr Özkan Ömer (n = 17), Dr Cetrulo Curtis L. (n = 15). (Fig. 8). These authors are affiliated with major academic centers involved in facial transplantation programs and represent key contributors to the advancement of VCA research. These authors represent the most productive contributors to the facial vascularized composite allotransplantation literature based on publication output. Their contributions encompass a broad range of clinical, experimental, translational, and review-based research and should not be interpreted as indicating direct participation in clinical face transplantation procedures. Co-authorship network analysis demonstrated a highly interconnected structure, with Dr Bohdan Pomahac positioned as a central hub linking multiple research clusters (Fig. 9). His network connects a wide range of collaborators across different institutions, reflecting a strong productivity on both clinical and translational research in the field. Similarly, Dr Eduardo D. Rodriguez forms a distinct but interconnected cluster, characterized by close collaboration within a defined group of co-authors, indicative of established institutional research teams. Additional clusters were identified around authors such as Dr Laurent Lantieri and Dr Alexandre G. Lellouch, representing important European contributions, as well as around Dr Maria Z. Siemionow, reflecting earlier pioneering work in facial transplantation. In the co-authorship network, node size is proportional to the number of publications, colors identify clusters of collaborating authors, and edge thickness reflects the strength of co-authorship relationships.

### Most cited publications

Citation analysis identified several highly influential articles in the field. The most cited publication was “Facial transplantation: the first 9 years” by Khalifian et al., with 308 citations.^14^ Other highly cited works included “First US Near-Total Human Face Transplantation: A Paradigm Shift for Massive Complex Injuries” by Siemionow et al. (214 citations) and “Face transplant: long-term follow-up and results of a prospective open study” by Lantieri et al. (174 citations)(15, 16). These studies represent key milestones in clinical and translational research within facial VCA.

### Keyword and research trend analysis

**Fig. 10:**
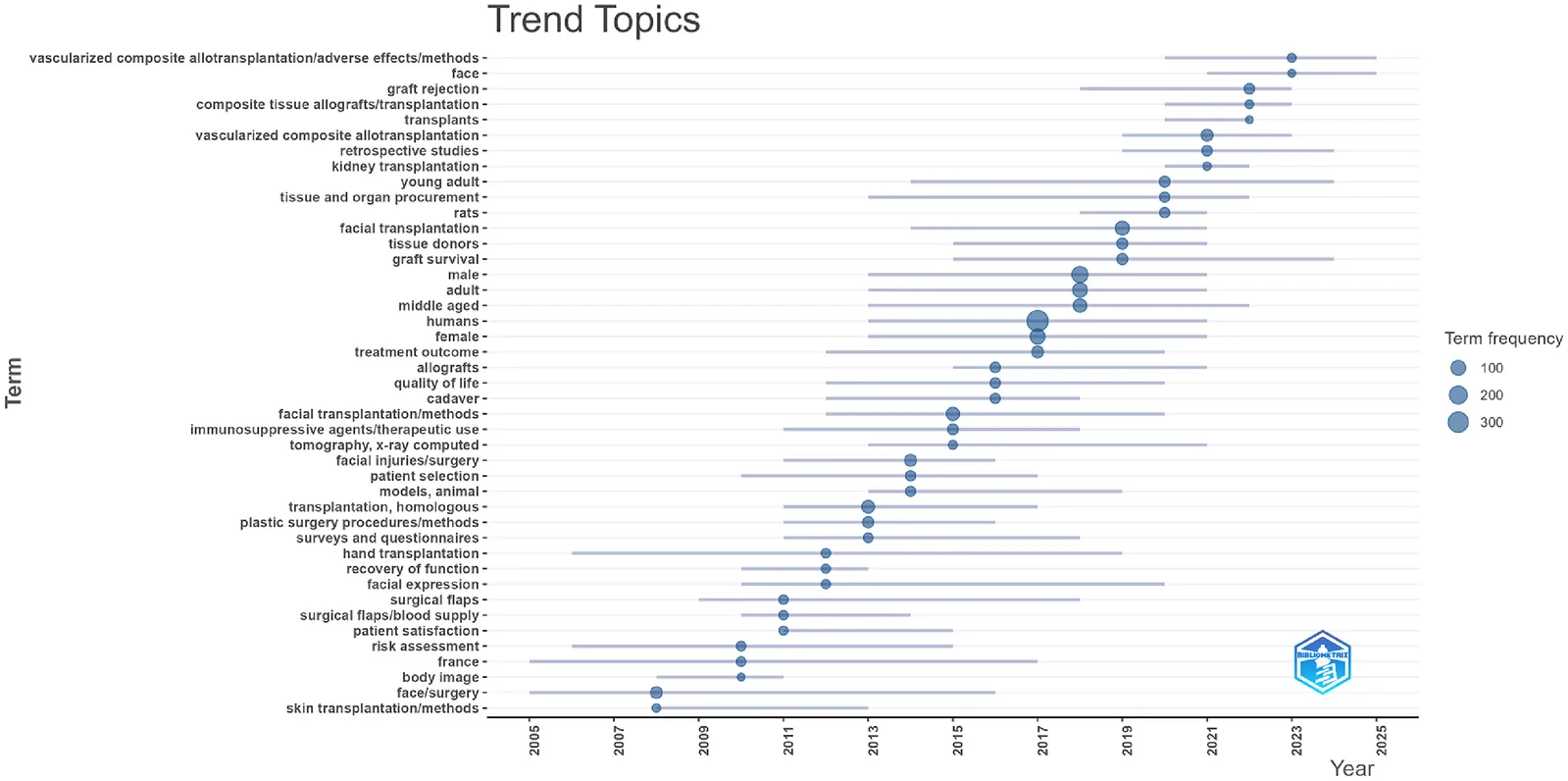
Trend topics illustrating the evolution of frequently used keywords in facial vascularized composite allotransplantation research.

Keyword co-occurrence analysis revealed major research themes centered on facial transplantation, immunosuppression, graft rejection, and reconstructive outcomes. Trend topic analysis further illustrated the temporal evolution of frequently used keywords in the literature (Fig. 10). Because the keyword analyses are generated from the author-provided keywords and indexed terminology of all publications retrieved by the predefined search strategy, the thematic maps also contain terms related to the broader field of vascularized composite allotransplantation. Consequently, keywords such as hand transplantation, upper extremity, or kidney transplantation may appear, reflecting publications that discuss facial transplantation within the wider context of VCA or compare facial transplantation with other transplant models. These terms should therefore be interpreted as contextual components of the retrieved literature rather than evidence that the search strategy extended beyond the intended focus on facial vascularized composite allotransplantation. Early research activity was primarily focused on surgical techniques, patient selection, and risk assessment, reflecting the initial development and clinical implementation of facial transplantation. Over time, there was a progressive shift toward outcomes research, including graft survival, functional recovery, and quality of life. In more recent years, a clear transition toward advanced and mechanistic research themes has emerged. Increasing attention has been directed toward antibody-mediated rejection, chronic rejection, and immunologic monitoring, alongside growing interest in biomarkers and molecular-level characterization of graft outcomes. Concurrently, technological innovations such as 3D printing, regenerative medicine, and tissue engineering have gained prominence. .^17^, ^18^, ^19^, ^20^, ^21^

### Emerging topics

**Fig. 11:**
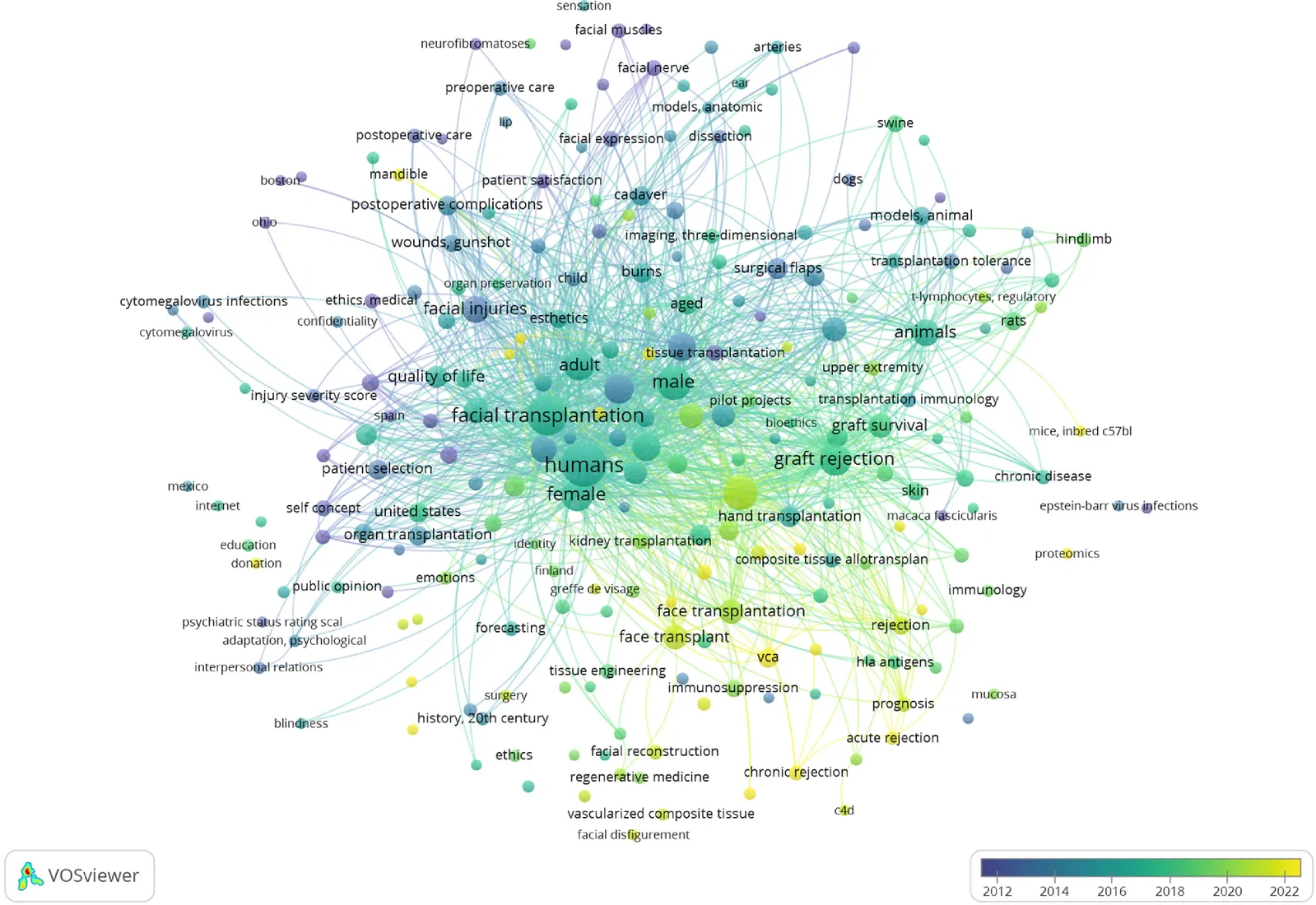
Temporal Keyword Co-occurrence Network Highlighting Evolving Research Themes in Facial VCA.

Beyond general keyword evolution, several recently emerging topics deserve particular attention because they reflect a shift in facial VCA research from descriptive clinical reporting toward mechanistic investigation of long-term graft injury (Fig. 11). In the keyword network, node size reflects keyword frequency, colors identify thematic clusters, and edge thickness represents the strength of keyword co-occurrence relationships. In recent years, the literature has increasingly focused on chronic rejection, antibody-mediated pathways, and advanced molecular profiling techniques. Among these, proteomic analyses, C4d and donor-specific antibody assessment, and experimental models of chronic rejection have become especially relevant, as they provide important insights into graft surveillance, pathophysiology, and future therapeutic targets.^22^, ^23^, ^24^, ^25^, ^26^, ^27^, ^28^

### Proteomic profiling and deep tissue immune mechanisms

Recent advances in immunoproteomic approaches have contributed to a deeper understanding of chronic rejection mechanisms in facial vascularized composite allotransplantation. In a study by Lee et al., digital spatial proteomic profiling was applied to a full-face graft explanted 88 months after transplantation, enabling detailed analysis of rejection at the tissue level.^22^ The investigation included 21 full-thickness samples, providing a comprehensive assessment of both superficial and deep structures within the graft. Quantitative proteomic analysis identified marked increases in proteins associated with cytotoxic T-cell and macrophage activity. Notably, expression levels of CD8 (6.67-fold increase, p = 0.0033), CD14 (4.77-fold increase, p = 0.0033), and CD163 (3.72-fold increase, p = 0.0224) were elevated, indicating sustained immune activation within the graft. In parallel, molecules involved in inflammatory and vascular signaling pathways, including indoleamine 2,3-dioxygenase 1 and STING, were also upregulated, suggesting engagement of innate immune and vascular injury pathways. Spatial and histologic analyses further demonstrated that a large proportion of deep graft vessels exhibited endothelial chimerism, with donor-derived CD8⁺ T cells localized adjacent to recipient-derived endothelial cells. This pattern of cellular interaction was associated with structural vascular changes consistent with arteriosclerotic remodeling. Together, these findings support the concept that chronic rejection in facial VCA involves immune-mediated injury of deep vascular structures, rather than being confined to superficial tissues. This may explain why conventional punch biopsies can underestimate the extent of graft pathology and highlights the need for more comprehensive diagnostic strategies targeting deeper tissue compartments.

### C4d deposition and donor-specific antibodies

Another fundamental focus in VCA research is the role of C4d deposition and donor-specific antibodies (DSA) in antibody-mediated rejection. In a recent systematic review, Huelsboemer et al. analyzed 22 studies including 26 patients (5 face and 21 hand transplant recipients) and evaluated 90 acute rejection episodes in which Banff grade, DSA status, and/or C4d staining were reported.^27^ Their analysis showed that rejection episodes with detectable DSA were associated with significantly greater severity, with a mean Banff grade of 2.7 ± 0.7 in DSA-positive episodes compared with 2.2 ± 0.6 in DSA-negative episodes (p = 0.005). These findings support a clinically meaningful relationship between DSA positivity and alloimmune activation. By contrast, the same review found that C4d positivity was not significantly associated with rejection severity (p = 0.33), suggesting that its diagnostic value in VCA remains uncertain. Among the reported episodes, C4d staining was assessed in 34 episodes, whereas both C4d and DSA were available in only 26 episodes, underscoring the limited and inconsistent reporting in the current literature. Importantly, no significant association was identified between concurrent C4d and DSA status (p = 0.23), further questioning whether C4d can reliably function as a surrogate marker of antibody-mediated injury in this setting.

These findings are consistent with broader observations summarized by Moris and Cendales et al., who reported that C4d staining in VCA is often inconsistent and may not reliably correlate with DSA presence, in contrast to its established role in solid organ transplantation.^29^ Collectively, these data suggest that while DSA monitoring represents a valuable biomarker of rejection risk, the role of C4d as a standalone diagnostic marker is limited. The absence of standardized criteria for antibody-mediated rejection in VCA further complicates interpretation, highlighting the need for integrated diagnostic strategies combining serologic, histologic, and molecular markers.

### Chronic rejection and allograft vasculopathy

Chronic rejection has emerged as a central challenge in long-term VCA outcomes, with increasing evidence highlighting its complex immunopathology. Experimental work by Fisher et al. developed novel murine hindlimb VCA models using defined MHC mismatch settings, including a single class II mismatch model (BM12 → C57BL/6 CD8 knockout recipients) designed to preferentially promote chronic rejection. These models closely replicate clinical features of chronic graft injury, including fibrosis, adnexal atrophy, mast cell infiltration, and progressive vascular remodeling.^26^ In the chronic BM12 model, which is characterized by a limited MHC class II disparity and reduced CD8⁺ T-cell–mediated cytotoxicity, >65% of grafts showed no overt macroscopic signs of rejection for up to 7 weeks post-transplantation. However, detailed histologic analysis at this time point revealed persistent inflammatory changes, fibrosis, and vascular alterations consistent with ongoing subclinical rejection, underscoring the insidious and progressive nature of chronic graft injury. Immunologic profiling demonstrated a significant expansion of activated B cells (B220⁺CD19⁺MHC II⁺CD138⁻) and plasma cells (B220⁺CD19⁺MHC II⁺CD138⁺) in both bone marrow and spleen compartments in chronic rejection models compared to acute rejection. This was associated with increased IgG-mediated donor-specific antibody activity, with higher levels observed in chronic models than in acute rejection settings. In parallel, circulating C4d levels, measured by ELISA, were significantly elevated in both chronic rejection models (chronic KO and chronic BM12) compared to syngeneic and acute controls, supporting a role for complement activation in late graft injury.

From a vascular perspective, chronic allograft vasculopathy was a consistent and defining feature. Histologic evaluation demonstrated intimal thickening (>25–50% luminal involvement), luminal narrowing, and intimal arteritis characterized by mononuclear cell infiltration beneath the endothelium. These features were observed in the majority of grafts in chronic rejection models, with approximately 75–80% of evaluable animals in both the chronic KO (BALB/c → C57BL/6 CD8 knockout) and chronic BM12 models demonstrating combined features of vasculopathy within 2 to 6 weeks post-transplantation, depending on the model. These vascular changes occurred under conditions of MHC mismatch and skewed immune responses toward humoral immunity (due to CD8⁺ T-cell deficiency and relative CD4⁺ T-cell–driven B-cell activation). Collectively, these structural vascular alterations were associated with impaired perfusion and progressive tissue damage, highlighting their central role in graft failure. Clinical observations by Krezdorn et al. further support these findings. In a cohort of 7 facial transplant recipients followed for up to 8 years, 3 patients developed histologic features of chronic rejection, including epidermal thinning, follicular damage, and dermal fibrosis.^30^ Molecular analysis revealed upregulation of AP-1 pathway components such as c-Fos and JunB, which are known regulators of collagen type I deposition and fibrotic remodeling. Notably, chronic rejection changes became clinically apparent only after prolonged follow-up, with a mean time to diagnosis of approximately 46 months, underscoring the delayed and progressive nature of the disease. Collectively, these data indicate that chronic rejection in VCA is a multifactorial process involving both cellular and humoral immune responses, progressive vascular injury, and tissue fibrosis. The integration of robust preclinical models with longitudinal clinical observations is essential to better define these mechanisms and to support the development of targeted therapeutic strategies.

## Discussion

Facial VCA represents a highly complex and evolving field at the intersection of reconstructive surgery and transplantation immunology. Over the past two decades, advances in microsurgical techniques and immunosuppressive strategies have enabled facial transplantation to become a viable option for select patients with severe facial disfigurement. However, long-term graft survival and functional durability remain limited by immunologic complications. The aim of this study was to characterize the global research landscape of facial VCA and to identify emerging scientific trends shaping the future of the field.

Our findings demonstrate a marked increase in scientific output over the last decade, reflecting growing clinical adoption and academic interest in facial transplantation. Research activity remains concentrated within a limited number of high-volume centers, with strong contributions from North America and Europe. The field is driven by a small group of highly productive authors and institutions, with specialized journals serving as the primary platforms for dissemination. Importantly, keyword and trend analyses reveal a shift toward mechanistic and translational research, particularly focusing on chronic rejection, antibody-mediated pathways, and advanced molecular profiling techniques.

These observations are consistent with previous reports in VCA and other emerging surgical fields, where innovation is typically driven by a limited number of specialized centers due to the technical complexity and resource requirements of the procedure.^31^ The predominance of North American and European institutions aligns with prior literature and reflects the concentration of established VCA programs.^32^^,^^33^ Beyond bibliometric patterns, the increasing focus on immunologic mechanisms parallels recent advances in the field. The most cited publications have played a pivotal role in shaping current understanding of facial transplantation. In particular, “Facial transplantation: the first 9 years” by Khalifian et al. synthesized early clinical experience and established foundational knowledge regarding indications, outcomes, and complications, thereby guiding subsequent clinical practice and research.^14^ Similarly, the report by Siemionow et al. on the first near-total face transplantation in the United States represented a major milestone, demonstrating the feasibility and reconstructive potential of complex VCA procedures.^15^ Long-term outcome data provided by Lantieri et al. further advanced the field by highlighting functional recovery and chronic complications, emphasizing the importance of longitudinal follow-up.^16^

More recent literature builds upon these foundational studies by focusing on mechanistic insights. For example, proteomic analyses by Lee et al. suggest that chronic rejection involves deep vascular injury not captured by conventional biopsy techniques, highlighting a critical limitation in current surveillance strategies.^22^ Similarly, some studies emphasize the evolving role of DSA and the limited reliability of C4d as a diagnostic marker, underscoring the lack of standardized criteria for antibody-mediated rejection in VCA.^27^^,^^29^ These findings are consistent with broader transplantation literature, where antibody-mediated injury and complement activation are recognized as major contributors to long-term graft failure, although their characterization in VCA remains incomplete.^34^ Furthermore, experimental models described by Fisher et al. and clinical observations by Krezdorn et al. highlight the multifactorial nature of chronic rejection, involving fibrosis, vascular remodeling, and immune dysregulation, with similarities to autoimmune fibrotic disorders.^26^^,^^30^ However, important discrepancies remain between studies, particularly regarding the relative contribution of cellular versus humoral mechanisms and the variability in histopathologic findings, which may be influenced by sampling limitations and the heterogeneity of VCA grafts. The limited number of clinical cases and the absence of standardized reporting frameworks continue to represent major challenges for the field.

From a clinical perspective, these findings have important implications for both patients and physicians. Facial VCA offers a unique opportunity for functional and psychosocial rehabilitation in patients with severe facial defects, but its long-term success is still constrained by the risk of chronic rejection and the need for lifelong immunosuppression.^35^, ^36^, ^37^, ^38^, ^39^ The identification of emerging trends such as advanced molecular profiling, biomarker development, and improved understanding of antibody-mediated mechanisms may contribute to earlier detection and more targeted management of graft injury.^40^ However, significant hurdles remain, including the lack of standardized diagnostic criteria, limited availability of specialized VCA centers, and the need for multidisciplinary expertise. Future progress will depend on the development of international registries, collaborative research networks, and high-quality longitudinal studies, as well as sustained funding to support innovation in immunologic monitoring and therapeutic strategies. Ultimately, improving long-term outcomes in facial VCA will require a combination of clinical expertise, translational research, and global collaboration.

This study has several limitations inherent to bibliometric analyses. First, the analysis was based solely on the PubMed database, which may not capture all relevant publications indexed in other databases such as Scopus or Web of Science, potentially leading to selection bias. In addition, only English-language publications were included. While this approach ensured standardized bibliographic metadata and reflected the language of the vast majority of internationally indexed facial VCA publications, it may have resulted in the exclusion of some relevant studies published in other languages. Consequently, the global scope of this study refers to the geographical origin and international distribution of research activity rather than comprehensive multilingual literature coverage. Second, bibliometric analyses rely on the metadata provided by the indexing database. Variations in the spelling or formatting of author names and institutional affiliations may not always be recognized automatically by bibliometric software, resulting in duplicate entities or fragmented collaboration networks. Similarly, inconsistencies in keyword reporting and indexing may influence network visualizations and productivity metrics. Although these limitations are inherent to bibliometric analyses and do not substantially alter the overall trends observed, they should be considered when interpreting the quantitative results. Third, citation counts may favor older publications and do not necessarily reflect current scientific impact or clinical relevance. Additionally, the relatively small number of clinical cases in facial VCA worldwide limits the overall volume of available literature and may influence the interpretation of research trends. Finally, although this study primarily reports bibliometric findings, selected recent publications are discussed to provide context for emerging research themes identified through the temporal keyword analysis. These examples are illustrative rather than systematically selected and should not be interpreted as a comprehensive or systematic synthesis of the available evidence. Consequently, this contextual discussion may be subject to post hoc selection bias and is intended solely to aid interpretation of the bibliometric trends.

## Conclusion

Facial vascularized composite allotransplantation is an evolving field characterized by rapidly expanding scientific output, concentrated expertise, and increasing interdisciplinary collaboration. This bibliometric analysis highlights the central role of leading institutions such as Harvard Medical School and key journals, including *Plastic and Reconstructive Surgery* in shaping the field. Foundational studies have established the clinical feasibility and long-term potential of facial transplantation, while recent research has shifted toward a deeper understanding of immunologic mechanisms, particularly antibody-mediated rejection, chronic rejection, and advanced molecular profiling. Emerging topics such as proteomics, biomarkers, and tissue engineering reflect a transition toward precision medicine and improved graft monitoring. Despite these advances, significant challenges remain, including limited case numbers, lack of standardized diagnostic criteria, and the burden of long-term immunosuppression. Future progress will depend on international collaboration, development of robust clinical registries, and integration of translational research to improve patient outcomes and expand the applicability of facial VCA.

## Statement of human and animal rights, or ethical

Not applicable.

## Informed consent

Not applicable.

## Financial disclosure statement

No funding.

## Declaration of competing interest

The remaining authors declare that the research was conducted in the absence of any commercial or financial relationships that could be construed as a potential conflict of interest.

## Appendix Supplementary materials

Image, application 1
