# Supplementary material for: Facial vascularized composite allotransplantation: Mapping global research trends and scientific impact
Source: JPRAS Open. 2026 Aug 19;52:169–81. doi: 10.1016/j.jpra.2026.08.004 (PMC13563230; doi:10.1016/j.jpra.2026.08.004)
Supplement: Supplementary file 1 [file mmc1.docx]

**Supplementary Material 1.** Research Strategy

| Database | Research String |
| --- | --- |
| Pubmed | (("face transplant"[tiab] OR "facial transplant"[tiab] OR "Face Transplantation"[MeSH]) OR (("vascularized composite allotransplant"[tiab] OR "composite tissue allotransplant"[tiab] OR VCA[tiab]) AND face[tiab])) |
